# Ocular Manifestation in Systemic Sclerosis—A Literature Review

**DOI:** 10.3390/life14050627

**Published:** 2024-05-13

**Authors:** Katarzyna Paczwa, Magdalena Rerych, Katarzyna Romanowska-Próchnicka, Radosław Różycki, Joanna Gołębiewska

**Affiliations:** 1 Opthalmology Department, Military Institute of Aviation Medicine, 01-755 Warsaw, Poland; kpaczwa@wiml.waw.pl (K.P.); mrerych@wiml.waw.pl (M.R.); rrozycki@wiml.waw.pl (R.R.); jgolebiewska@wiml.waw.pl (J.G.); 2 Department and Polyclinic of Systemic Connective Tissue Diseases, National Institute of Geriatrics, Rheumatology and Rehabilitation, 02-637 Warsaw, Poland

**Keywords:** systemic sclerosis, ocular manifestation, scleroderma, ocular involvement, retina, dry eye syndrome

## Abstract

Systemic sclerosis (SSc) is a chronic autoimmune connective tissue disease that affects more than 2 million people worldwide. It manifests through vasculopathy, an abnormal immunological response, and fibrosis leading to dysfunction of the multiple organs. The disease is categorized into two subtypes: limited cutaneous SSc and diffuse cutaneous SSc. Scleroderma can affect vital organs with respiratory, cardiac, renal, ocular, and dermatological complications. The ocular manifestations of the disease can occur in the anterior and posterior segments of the eye. Changes in the anterior segment related to the disease include eyelid skin remodeling, dry eye syndrome, and conjunctival abnormalities. The disease’s impact on the posterior segment of the eye mostly causes pathologies in the retinal microcirculatory system and abnormalities in the optic nerve. This review provides detailed insights into ocular complications associated with scleroderma.

## 1. Introduction

Systemic sclerosis (SSc) is a rare and complex autoimmune disease that manifests itself through vasculopathy, an abnormal immunological response, and fibrosis, leading to progressive dysfunction of multiple organs [1,2]. It affects about 2.5 million people worldwide and is more common among women [1,2]. The intricate interplay of these pathological processes significantly impacts vital organs such as the lungs, heart, kidneys, eyes, and skin [3]. The cumulative effects not only compromise the quality of life but also pose a serious threat to the life expectancy of individuals affected by this condition.

**Figure 1:**
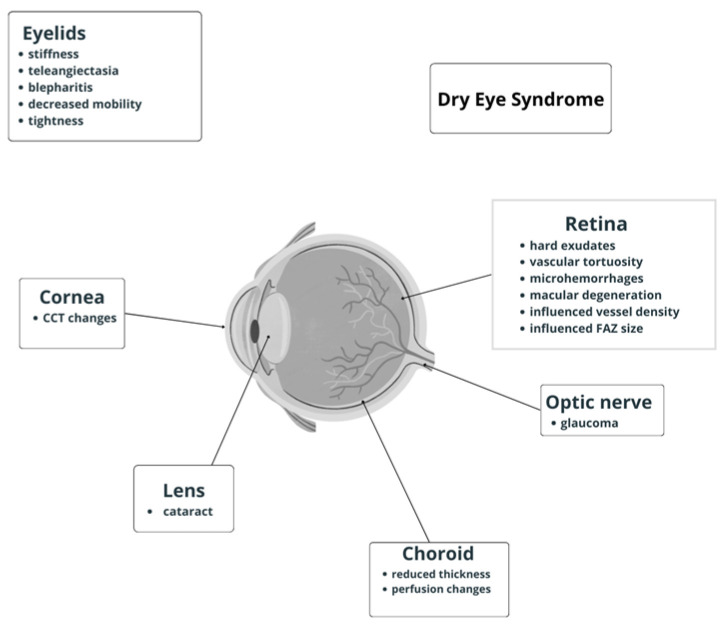
Schematic diagram of ocular manifestations in systemic sclerosis.

The disease is categorized into two subtypes: limited cutaneous SSc (lcSSc) and diffuse cutaneous SSc (dcSSc) [4,5]. These distinctions are crucial in understanding the varied clinical manifestations and disease trajectories. In limited cutaneous SSc, the fibrotic changes predominantly affect the skin of the hands, face, and lower arms. Conversely, diffuse cutaneous SSc is characterized by widespread skin fibrosis, affecting large areas of the body. This subtype often entails a more aggressive course, with rapid progression of fibrosis not only in the skin but also in internal organs, leading to more severe and widespread organ dysfunction [6]. The impact of SSc on vital organs is profound, with respiratory, cardiac, renal, gastrointestinal, musculoskeletal, ocular, and dermatological complications. Lung involvement, in particular, can result in pulmonary fibrosis, making breathing difficult and compromising oxygen exchange. Cardiac complications may include heart inflammation and scarring, leading to impaired function. Renal complications can manifest as hypertension and renal crisis, affecting kidney function. A wide variety of ocular manifestations have been reported in SSc patients, and because of the disease rarity, most data came from case reports or small case studies. The frequent eye involvement in SSc depends on the high collagen content in the ocular tissues and the presence of a microcirculatory system in the retina, making the eye susceptible to pathological changes. Fibrosis impact is mostly observed in the anterior segment structures, while vasculopathy affects retinal and choroidal microcirculation. This review provides detailed insights into ocular complications associated with scleroderma. Regular ophthalmic examinations are crucial for the early detection and management of ocular manifestations in scleroderma as timely intervention can help alleviate symptoms and prevent complications that may result from both skin and ocular involvement (as presented in Figure 1).

## 2. Methodology 

The objective of the literature review was to identify and evaluate the relevant studies published on ocular manifestations in systemic sclerosis. A manual search was conducted through the major electronic databases (PubMed, Google Scholar) using the following search terms: ‘systemic sclerosis’, ‘ocular manifestation’, ‘ocular involvement’, and ‘scleroderma’ in different combinations. The results formed a list of potentially relevant articles, with a focus on the most recent publications. A total of 37 compatible research publications were identified and used to compile this review.

## 3. Anterior Segment of the Eye

### 3.1. Eyelids

**Figure 2:**
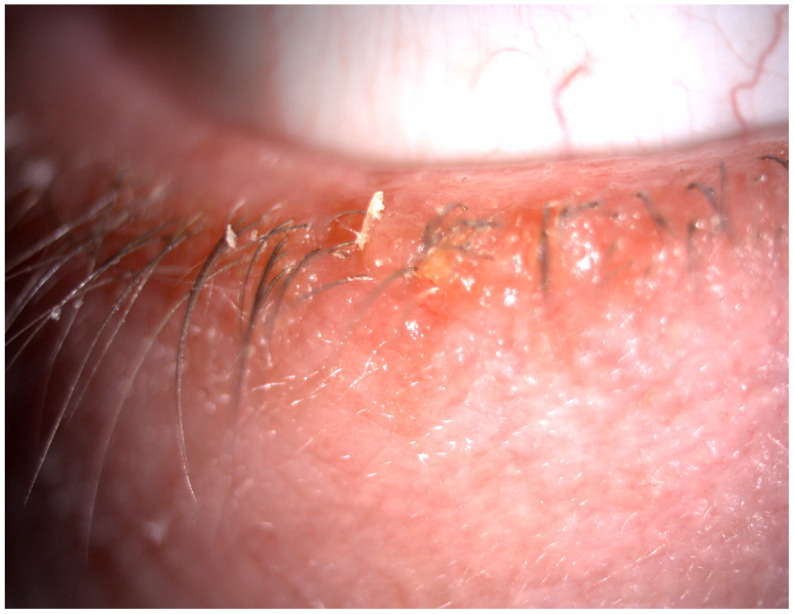
Blepharitis in a scleroderma patient.

**Figure 3:**
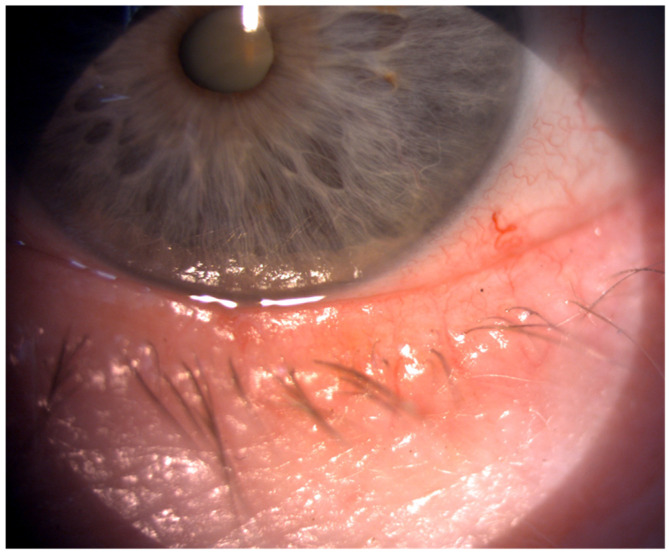
Telangiectasia of the eyelid skin.

The eyelids protect the ocular surface and play a crucial role in adequate tear film distribution and tear drainage. They are considered the thinnest layer of skin in the body. The studies showed that eyelid lesions are the most common ocular manifestation of scleroderma, both in limited and diffuse forms of the disease [5,7]. Disturbance in fibroblast proliferation leads to the accumulation of the extracellular matrix (mostly type I collagen) in the skin [5]. The fibrosis in the eyelid skin can result in its stiffness, tightness, and lagophthalmos [5,8], which cause decreased eyelid mobility and consequently disturbances in spreading tear film over the ocular surface. Szucs et al. [9], in their study, investigated 102 eyes of 51 SSc patients. They observed pathologies in the eyelids in more than 56% of the study group. Stiffness, tightness, telangiectasia, and blepharitis were the most common findings [9], as shown in Figure 2 and Figure 3.

Furthermore, similar results were presented by Sahin et al. [5], who examined skin thickening using a modified Rodnan skin score (mRSS). The mRRS is an objective method for assessing skin involvement—the higher the score the worse prognosis of the disease. Their results divided patients into three groups: 32% of patients revealed mild, 52% moderate, and 16% severe thickening of the skin [5]. Those pathological changes in eyelid structures can result in lagophthalmos and ptosis. Gomes et al. [7] reported that eyelid abnormalities were more common in patients with diffuse scleroderma and more frequent in younger patients with early-onset disease. These eyelid abnormalities consequently lead to the impairment of tear film distribution and dry eye syndrome occurrence. Moreover, Atik et al. [5] in their study discovered that scleroderma patients with moderate and severe eyelid skin thickening had lower anterior chamber depth (ACD) measurements than healthy controls. However, anterior movement of the lens diaphragm causing shallowing of the anterior chamber may be due to fibrosis in the ciliary body rather than mechanical obstruction caused by thickening of the eyelid skin [5].

### 3.2. Dry Eye Disease

**Figure 4:**
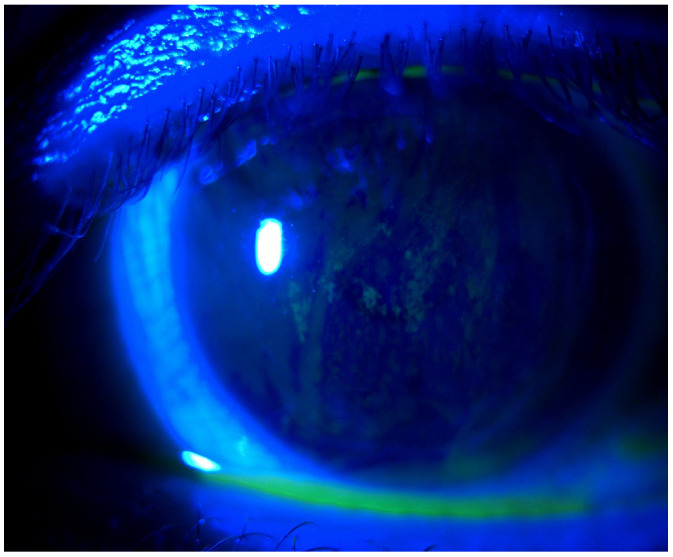
Photo of the corneal abrasions after fluorescein staining.

Dry eye disease (DED), also known as keratoconjunctivitis sicca, is a complex disorder characterized by the loss of tear film homeostasis that causes mild to severe irritation [10]. The condition affects several hundred million people worldwide [11]. Symptoms related to dry eye are one of the most common ocular findings among SSc patients. The tear film is a complex structure responsible for lubricating, nourishing, and protecting the ocular surface. It is divided into three layers: inner mucin, middle aqueous, and outer lipid layer. The mucus layer is mostly secreted by the conjunctival goblet cells, the aqueous fluid by the lacrimal glands, and the lipid layer by the meibomian glands. One of the studies revealed that in systemic sclerosis, lacrimal gland fibrosis causes decreased production of aqueous tears. Moreover, the reduced number of conjunctival goblet cells leads to a reduction of mucin layer secretion [12]. Consequently, these factors causing the reduction of tear secretion lead to dry eye symptoms. In addition, meibomian gland dysfunction and chronic blepharitis often occurring among SSc patients could increase symptoms of DED [13] (see Figure 4).

Depending on the study the prevalence of systemic sclerosis patients with DED varies from 37% to 79% [7,12]. For example, Gomes et al. presented the results of ocular complications in 45 SSc patients. In their study, dry eye evaluation was performed using tear breakup time (TBUT), Schirmer I test, and rose bengal staining. The authors reported that DED and eyelid skin abnormalities were the most common findings and DED was observed in 22 patients (48.9 %) [7]. Wangkaew et al. also found a higher prevalence of DED in SSc patients (54%) compared with the healthy control group (16%) (*p* < 0.01) [14]. Similar results were reported by Laovirojjanakul et al. [12] who evaluated 84 patients with scleroderma, including 25 with a limited and 59 with a diffuse form of the disease. In their study, they used the questionnaire—the Ocular Surface Disease Index (OSDI)—and objective tests such as the Schirmer I test, TBUT, and ocular surface staining. Their results showed that 52.38% of SSc patients suffered from DED. They claimed that there were no significant differences in dry eye test results in SSc types. Moreover, the mean age and the median disease duration in DED patients were higher than in those without DED (*p* = 0.004 and 0 = 0.019). Szucs et al. [9], in their study, had examined 51 patients and found DED in 64.71% of them. They revealed a statistically significant correlation between OSDI and TBUT test results and nailfold capillaroscopy findings. Furthermore, dry eye symptoms in SSc can be caused by Sjogren’s syndrome (SS) associated with systemic sclerosis because of lymphocytic infiltration of the main lacrimal gland causing its destruction [15]. Sjogren’s syndrome occurs in 17–29% of scleroderma patients [15]. Salliot et al. [16] conducted a study to determine whether SS coinciding with systemic sclerosis is secondary to SSc or related to SSc. SS associated with autoimmune diseases is less severe with a lower frequency of anti-SSA/SSB antibodies than primary SS. The results of their study determined that SS is mostly related to, rather than secondary to, SSc. Moreover, they claimed that patients diagnosed with Sjogren’s syndrome with SSc were more predisposed to other autoimmune diseases.

### 3.3. Conjunctiva

Conjunctiva seems to be vulnerable to scleroderma vasculopathy because of its high vascularization. Some studies presented conjunctival abnormalities in scleroderma. Szucs et al. [9] examined 102 eyes of 51 SSc patients to assess ocular symptoms and reported two main pathologies: blood vessel congestion and the loss of fine vessels in over 15 % of the study group. Their results were similar to previous findings regarding the conjunctiva [7,17]. Furthermore, West et al. [17], in their research, described that these abnormalities resemble changes in nailfold capillaries visualized in capillaroscopy. In another study, Mencel et al. [18] reported results on conjunctival biopsies of 21 scleroderma patients. Microscopic sample examination revealed the loss of goblet cells, the thinning and the keratinization of the conjunctival epithelium, and mast cell infiltration.

### 3.4. Cornea

The cornea is a transparent avascular tissue that contributes two-thirds of the refractive power of the eye. The current literature offers a few publications on corneal changes in systemic sclerosis. Due to its high collagen content (type I and V), the cornea is vulnerable to remodeling changes [9]. However, the results of the studies are controversial. Emre et al. [19] included the right eyes of 29 SSc participants to evaluate corneal biomechanical parameters by the Reichert ocular response analyzer (ORA) consisting of corneal hysteresis, corneal resistance factor (CRF), and intraocular pressure (IOP). Their results revealed higher mean CRF and IOP values compared with healthy controls. In another study, Gomes et al. [20] correlated central corneal thickness (CCT) in 37 SSc patients with healthy individuals. Their results did not reveal a significant difference between the two groups in the right (*p* = 0.83) and left eye (*p* = 0.67). Similar results were reported by other authors [5]. However, in a previous study, Serup et al. [21] reported an increased CCT in SSc patients compared with the control group (*p* < 0.001). They noted that corneal thickening usually occurs in the early stages of the disease, especially during the first 8 years. Moreover, Waszczykowska et al. [22], in their study, revealed a higher incidence of astigmatism among SSc patients compared with healthy participants (*p* < 0.01). This refractive error was observed in over half of the patients’ eyes in the study group. On the other hand, Sahin et al. [23] found that CCT was decreased in scleroderma. Szalai et al. [24], in their study, examined 33 patients with SSc using in vivo confocal microscopy to evaluate corneal microstructure. Their results demonstrated that keratocyte cell densities in the anterior stroma were lower than in the healthy subjects (*p* < 0.0001). They also found significantly lower subbasal nerve fiber parameters in the study group compared with the controls (*p* < 0.05). In addition, Anayol, et al. [25] presented a case of keratoconus associated with the disease. Therefore, further study is needed to determine the SSc effect on the cornea.

### 3.5. Iris

Iris transillumination defects were the most common changes found in studies on iris pathologies in SSc patients. Gomes et al. [7] reported this pathology in 8.9% of their subjects. Moreover, other abnormalities such as irregular iris, atrophy, and distorted blood vessels were mentioned in the literature [9].

### 3.6. Pupil Diameter

The studies indicate that the autonomic nervous system could be involved in SSc, especially in Raynaud’s phenomenon, which is usually an initial symptom of the disease [26]. The authors revealed an increased activity in the sympathetic nervous system and a decreased activity in the parasympathetic nervous system. Bertinotti and del Roso [26], in their study, compared a pupil diameter in 44 SSc patients with that in 40 healthy controls and examined the impact of the substance *p* (a direct sphincter pupillae muscle constrictor). In scleroderma patients, the rested pupil diameter was smaller in comparison with the control group (*p* < 0.001). Moreover, substance P induced more increased myosis in the study group than in healthy subjects. These changes were more intense in the lSSs group. On the other hand, Waszczykowska et al. [22] did not find any pupil abnormalities in their study.

### 3.7. Cataract

A cataract is a hardening and clouding of the natural crystalline lens, which may result in progressive visual impairment. In most people, cataract symptoms usually occur in patients aged 60 and older. However, it may appear in younger patients after trauma, long corticosteroid treatment, or diabetes. In the study by Szucs et al. [9], who examined patients with SSc with a mean age of 65.39 (SD ± 10.09) years, 50.98% had cataracts. In that study, pseudophakic intraocular lenses had already been implanted in 15.38% of the patient’s eyes and another 35.6% of them had cataracts [9]. Similar results were reported by Gomes et al. [7] who found cataracts in 42.2% of the patients. The age of the study population ranged from 22 to 77 years, with an average age of 51 years. Moreover, 89.5% of them were currently taking or had previously been taking systemic corticosteroids, compared with 60% of the patients without cataracts (*p* = 0.04) [7]. Current research does not allow us to clearly determine the impact of systemic sclerosis on cataract development. Most of them showed a significant association between steroid exposure and the occurrence of cataracts in SSc patients. However, it should be emphasized that the latest therapeutical recommendations for the treatment of systemic sclerosis restrict the application of glucocorticoids because of adverse effects on skin lesions and the risk of SRC. Most cataracts are related to age and secondly to corticosteroid treatment [5,9,27].

## 4. Posterior Segment of the Eye

### 4.1. Glaucoma

Glaucoma is a progressive chronic optic neuropathy resulting in irreversible vision loss worldwide. It is characterized by progressive retinal ganglion cell death and optic nerve head changes. The pathogenesis of glaucoma remains poorly understood. It has been postulated that vascular pathologies may play a significant role in the development and progression of this condition.

Szucs et al. [9] reported their results of glaucoma in patients with systemic sclerosis. Glaucoma was diagnosed in 21.57% of them. Different types of glaucoma were presented: open-angle glaucoma, closed-angle glaucoma, pigmentary, and normal tension glaucoma. Similar results were reported by Gomes et al. who enrolled 31 patients with SSc and 23% of them had glaucoma. None of them had a history of steroid administration. In the study, they evaluated the correlation between glaucoma presence and a pattern of nailfold capillaroscopy (NC). They divided the abnormalities observed in NC into two groups: mild and severe. The mild group was defined as the presence of giant capillaries, microhemorrhage, and relatively well-preserved capillary distribution. Severe findings included the loss of capillaries and the development of the avascular areas with the absence of the giant capillaries. 

The results of their study did not reveal a significant correlation between glaucoma diagnosis and the NC pattern (*p* = 0.86) [28]. Gomes et al. [7], in another study, confirmed the prevalence of glaucoma in 13.3% of patients with SSc. In total, 66.7% of them were taking or had previously taken systemic corticosteroids, compared with 75.5 % of subjects without glaucoma (*p* = 0.64) [7]. Further studies are needed to improve the understanding of glaucoma in patients with systemic sclerosis. 

**Figure 5:**
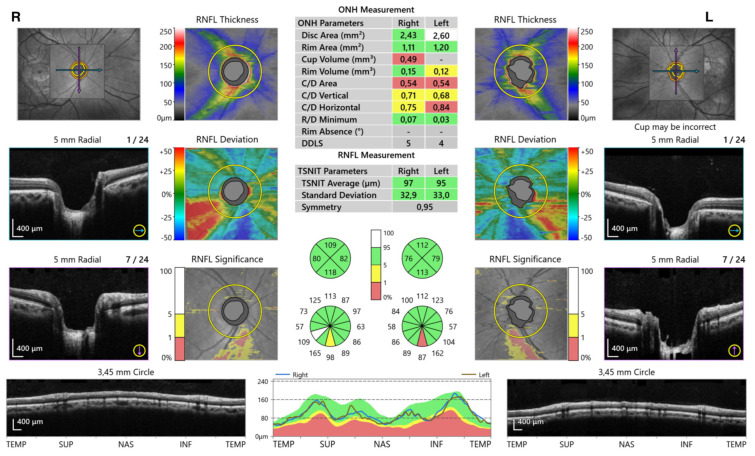
OCT report of a scleroderma patient shows the results of RNFL thickness and optic disc morphology analysis.

Moreover, Sahin-Atik et al. [29] analyzed retinal nerve fiber layer (RNFL) and optic disc morphology in a 28-year-old patient with SSc diagnosed using optical coherence tomography (OCT) (see Figure 5).

There was no significant difference in the average and 4-quadrant RNFL thickness between the patients and the control group. In patients who had a cup-to-disc ratio (c/d) > 0.5. inferior quadrant RNFL thickness was significantly thinner (*p* < 0.05). Optic disc morphology measurements were similar in both groups. It might be assumed that the results are related to the disease duration and further studies are needed to identify the long-term glaucomatous optic neuropathy in SSc [29].

### 4.2. Uveitis

Uveitis is well known as being associated with systemic autoimmune diseases, including systemic lupus and negative spondyloarthropathies. However, the literature indicates that uveitis in scleroderma mostly occurs in a juvenile form. Bolad et al. [30] presented the case of an adult man with systemic sclerosis who developed bilateral anterior uveitis. In another study, Waszczykowska et al. [22] examined 27 SSc patients and only 1 of them had a history of uveitis. A few studies reported cases of uveitis in lSSc. The most recent one, published by Khalayli et al. [31] presented a case of recurrent unilateral uveitis in the course of limited scleroderma.

### 4.3. Vitreous Body

The literature on vitreous body examination in SSc patients indicates that the observed changes are related to age rather than the disease, including vitreous syneresis and collapse [17].

### 4.4. Retina and Choroid

The choroid is a vascular structure of the eye that provides oxygen and nutrients to the outer retinal layers. This is one of the most vascularized tissues in the body, responsible for over 70% of the blood flow in the eye. Microvascular changes in SSc primarily tend to affect small capillaries and arterioles. In rheumatology, peripheral microcirculation abnormalities of nailfold capillaries observed in NC are used in everyday practice to diagnose and monitor the disease.

**Figure 6:**
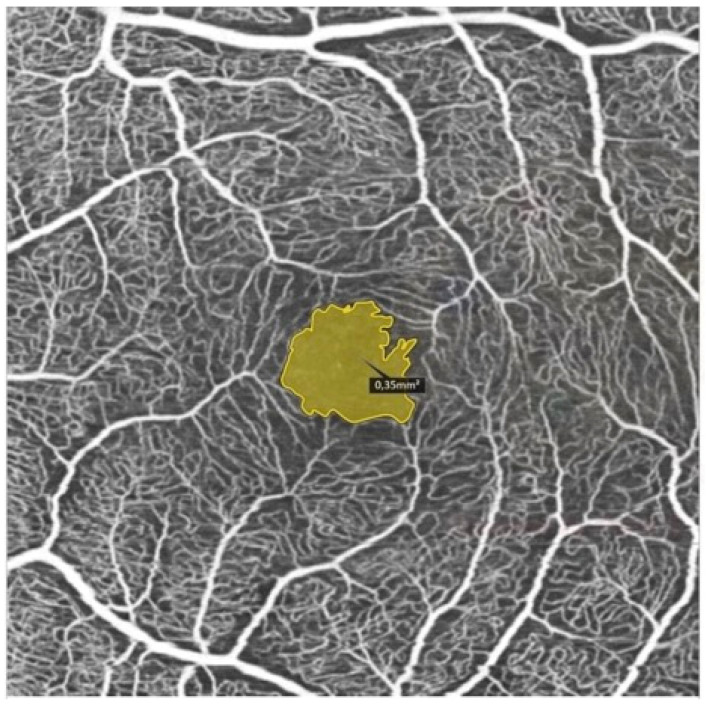
FAZ area measurement using OCTA in a patient with systemic sclerosis.

Therefore, generalized vasculopathy in the course of systemic sclerosis plays an important role in both retinal and choroidal perfusion disorders. Scleroderma is characterized by vascular insufficiency caused by a reduction in the number of capillaries and the narrowing of small arteries, arterioles, and capillaries. Refs. [32,33] Using noninvasive OCT angiography (OCTA), we are able to examine retinal microvasculature and its correlation with vasculopathy observed in the peripheral microcirculation. Recent studies focused on retinal findings in scleroderma. In a previous study, Ushiyama et al. [34] evaluated the fundus of 29 SSc patients and compared them with 38 healthy controls. Their results showed a higher incidence of retinal pathologies compared with the control group (*p* = 0.011), including hard exudates, vascular tortuosity, microhemorrhage, and macular degeneration. They analyzed the correlation between the detected retinal abnormalities and the patterns observed in the nailfold capillaroscopy in the study group. However, no significant correlation was found. Similar results regarding retinal vessels observed in fundus examination were presented by Waszczykowska et al. [22], who reported stenoses and increased tortuosity in SSc. In their study of 29 patients with SSc, Serup et al. [35] described changes in the late angiographic phase in 33% of the study group. Furthermore, Hekimsoy et al. [32] investigated the retinal and choroidal microvasculature using OCTA. They included 45 participants with systemic sclerosis without clinical evidence of retinopathy. Their results showed significantly lower superficial capillary plexus vessel density of the whole image, fovea, parafovea, and perifovea, and the deep capillary plexus vessel density of the fovea (*p* < 0.05). Hekimsoy et al. [32] noticed no statistically significant difference in the foveal avascular zone (FAZ) area, FAZ perimeter, acircularity index of FAZ, foveal density-300, and choriocapillaris flow area measurements between patients and the control group (see Figure 6).

**Figure 7:**
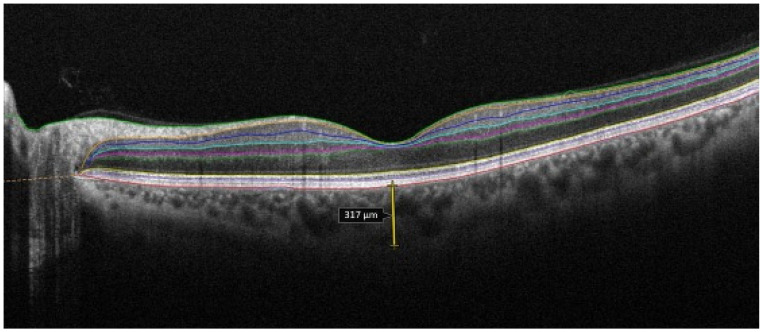
Choroidal thickness measurement using OCT in a patient with scleroderma.

They also found no significant difference in mean optical coherence tomography angiography measurements between two subtypes: limited and diffuse systemic sclerosis [32]. Aydin et al. [36] investigated the morphological changes in the fovea and choroid in scleroderma patients and healthy subjects. There was no difference in the mean central foveal thickness and subfoveal choroidal thickness values in patients with scleroderma compared with the controls (*p* > 0.05). In that study, Aydin et al. did not find significant differences in mean central foveal thickness and choroidal thickness of both eyes comparing lcSSc with dSSc subtypes [36] (see Figure 7).

In another study of 46 scleroderma patients, Coskun et al. [33] demonstrated significantly thinner nasal (*p* = 0.012), temporal (*p* = 0.046), and subfoveal (*p* < 0.001) choroid compared with the controls. The fundus examination revealed local RPE changes in 15% of the eyes as the result of damage to the choroidal vasculature at the choriocapillary level. Kok et al. [37] demonstrated the direct effects of SSc on retinal and choroidal microvasculature in patients without hypertension. They found increased vascular tortuosity, focal or general arteriolar narrowing, arteriovenous notch, severe exudation, microhemorrhage, and pigment epithelial changes in the retina in fundus examination. They diagnosed retinopathy in patients with at least two of those abnormalities. Those symptoms might resemble secondary changes caused by hypertension, which is detected in up to 50% of SSc patients. Kok et al. [37] showed an increase in the outer retina 1–3 mm flow area and a decrease in both superficial (*p* = 0.003) and deep (*p* = 0.001) capillary plexuses vessel density, using OCTA. The FAZ area in the superficial retinal capillary plexus was significantly larger (*p* = 0.01) in subjects with SSc, and the FAZ in the deep retinal capillary plexus did not differ significantly compared with the healthy control group (*p* = 0.01). Compared with other studies, the authors also observed a decrease in subfoveal choroidal thickness in the SSc group. [37] Rommel et al. [38] evaluated 30 eyes of the patients with SSc and 30 eyes of the healthy controls. In the first group, the study showed reduced macular volume (*p* = 0.017). The same patients revealed decreased Sattler’s layer and Haller’s layer thickness. The perfusion of both retinal plexuses as well as Sattler’s and Haller’s layers were significantly reduced in the SSc group. In the early stages of SSc, no correlation was found between perfusion of the retina and choroid compared with healthy controls. However, as the disease progressed, the reduction in perfusion became statistically significant [38]. On the other hand, Busquets et al. described three patients with systemic sclerosis who developed acute retinal occlusion as a manifestation of the retinal vessel fibroproliferative vasculopathy [39]. Furthermore, Mihailovic et al. [40], in their study, demonstrated a statistically significant correlation between capillary density obtained in NC and vessel density of the choriocapillaris (*p* = 0.011), as well as a significant negative correlation between the mRSS and the vessel density in OCTA. Similar results were demonstrated by Cutolo et al. [41] who examined 32 SSc patients. Their results revealed a significant correlation between the mean capillary number observed in NC with the retinal and choroidal perfusion in OCTA (*p* < 0.05). In our previous study, we observed a correlation between FAZ size and scleroderma patterns [42]. In conclusion, optical coherence tomography angiography is able to reveal early retinal and choroidal microvascular changes in patients with SSc, even without clinical evidence of retinopathy. The retinal and choroidal circulation may play a significant role in the assessment of generalized arteriolar and capillary pathologies in systemic sclerosis [32,33,35,37].

## 5. Conclusions

A review of the published literature reveals that ocular lesions are commonly observed in individuals with systemic sclerosis (SSc). The most prevalent ocular pathologies in this context are eyelid fibrosis, dry eye disease, and retinal abnormalities. However, there is no clear correlation between these ocular changes and the progression of SSc. Further studies involving a larger number of subjects are necessary to determine the relationship between ophthalmological changes and the stages and type of SSc, as well as the disease parameters and nailfold capillaroscopy.

## Data Availability

Data are contained within the article.
